# A Prospective Pilot Trial for Pallidal Deep Brain Stimulation in Huntington’s Disease

**DOI:** 10.3389/fneur.2015.00177

**Published:** 2015-08-18

**Authors:** Lars Wojtecki, Stefan J. Groiss, Stefano Ferrea, Saskia Elben, Christian J. Hartmann, Stephen B. Dunnett, Anne Rosser, Carsten Saft, Martin Südmeyer, Christian Ohmann, Alfons Schnitzler, Jan Vesper, for the Surgical Approaches Working Group of the European Huntington’s Disease Network (EHDN)

**Affiliations:** ^1^Department of Neurology, Centre for Movement Disorders and Neuromodulation, Medical Faculty, Heinrich-Heine-University Düsseldorf, Düsseldorf, Germany; ^2^Institute of Clinical Neuroscience and Medical Psychology, Medical Faculty, Heinrich- Heine-University Düsseldorf, Düsseldorf, Germany; ^3^Brain Repair Group, School of Biosciences, Cardiff University, Cardiff, UK; ^4^Department of Neurology, St. Josef-Hospital, Ruhr University, Bochum, Germany; ^5^Coordinating Centre for Clinical Trials, Heinrich-Heine-University Düsseldorf, Düsseldorf, Germany; ^6^Department of Stereotactic and Functional Neurosurgery, Medical Faculty, Heinrich-Heine-University Düsseldorf, Düsseldorf, Germany; ^†^Group members are listed in the Author Contributions section

**Keywords:** Huntington’s disease, deep brain stimulation, chorea, pallidum

## Abstract

**Background:**

Movement disorders in Huntington’s disease are often medically refractive. The aim of the trial was assessment of procedure safety of deep brain stimulation, equality of internal- and external-pallidal stimulation and efficacy followed-up for 6 months in a prospective pilot trial.

**Methods:**

In a controlled double-blind phase six patients (four chorea-dominant, two Westphal-variant) with predominant movement disorder were randomly assigned to either the sequence of 6-week internal- or 6-week external-pallidal stimulation, or vice versa, followed by further 3 months chronic pallidal stimulation at the target with best effect-side-effect ratio. Primary endpoints were changes in the Unified Huntington’s Disease Rating Scale motor-score, chorea subscore, and total motor-score 4 (blinded-video ratings), comparing internal- versus external-pallidal stimulation, and 6 months versus baseline. Secondary endpoints assessed scores on dystonia, hypokinesia, cognition, mood, functionality/disability, and quality-of-life.

**Results:**

Intention-to-treat analysis of all patients (*n* = 3 in each treatment sequence): Both targets were equal in terms of efficacy. Chorea subscores decreased significantly over 6 months (−5.3 (60.2%), *p* = 0.037). Effects on dystonia were not significant over the group due to it consisting of three responders (>50% improvement) and three non-responders. Westphal patients did not improve. Cognition was stable. Mood and some functionality/disability and quality-of-life scores improved significantly. Eight adverse events and two additional serious adverse events – mostly internal-pallidal stimulation-related – resolved without sequalae. No procedure-related complications occurred.

**Conclusion:**

Pallidal deep brain stimulation was demonstrated to be a safe treatment option for the reduction of chorea in Huntington’s disease. Their effects on chorea and dystonia and on quality-of-life should be examined in larger controlled trials.

## Introduction

Huntington’s disease (HD) is a progressive, motor, cognitive, and psychiatric neurodegenerative disorder caused by an expanded CAG repeat in the Huntingtin gene. To date, there is no causal or disease modifying treatment for HD. The typical motor symptom in HD is chorea, but other movement abnormalities, such as dystonia and hypokinesia, can occur – especially in juvenile onset HD (Westphal variant). Although dopamine antagonists and dopamine-depleting drugs have demonstrated some symptomatic efficacy in patients in whom chorea is the dominant feature, they often do not produce significant functional improvement. The rationale for using deep brain stimulation (DBS) of the internal pallidum (GPI) for HD is based on evidence that GPI–DBS is effective in suppressing non-HD choreiform dyskinesias, such as levodopa-induced dyskinesia (1) in Parkinson’s disease and the dystonic movements of primary dystonia (2). Case reports have shown that GPI–DBS has been effective in various other neurological disorders presenting with choreiform symptoms [for review, see Ref. (3)]. For the treatment of HD-chorea itself, several reports provide preliminary evidence for the feasibility of pallidal DBS, with reports up to 5 years (4–6). There might be a better response on chorea rather than on dystonic symptoms (7). The experience with for Westphal patients is sparse (8). Some case reports on HD-chorea used blinded assessments (9, 10) but the only prospective two clinical trials evaluating DBS for choreatic movements (besides levodopa-induced dyskinesias in Parkinson’s disease) was performed for dystonia–choreoathetosis in cerebral palsy (11) and recently for HD (12) with the latter however lacking a destinct pre-defined protocol, a control-group, blindend assessments, and systematic evaluation of adverse events.

It is not known whether high frequency internal pallidal stimulation affects cognitive abilities in HD. Most reports do not identify any changes, however, some decline was noted in two patients (13). In contrast, motor *and* cognitive improvements were reported with stimulation of the external pallidum (GPE) in animal experiments (14). Further evidence for the usefulness of GPE stimulation comes from preliminary PET imaging data (15) of a series of HD patients undergoing GPE–DBS, which showed decreasing activity and modulation of connectivity within the basal ganglia-thalamocortical circuit and sensorimotor cortical areas.

Given this scientific background, it is legitimate to assess pallidal DBS as a treatment option in HD, starting with a prospective clinical trial assessing its safety and efficacy. About efficacy, in this prospective 6-months pilot trial, we tested the hypothesis that randomized GPI and GPE stimulation would be equivalent in terms of their effects on motor function. We also tested the hypothesis that chronic stimulation of the pallidum would be a safe and effective treatment first in motor function as chorea, hypokinesia and dystonia and, second on non-motor aspects as cognition, emotion, functional disability and quality of life.

## Patients and Methods

The trial was designed as a prospective pilot trial focusing on the safety and efficacy of pallidal DBS in HD. The protocol consisted of a randomized, controlled crossover design to examine the hypothesis of the equivalence of GPI and GPE stimulation, and an uncontrolled 6-month follow-up to assess chronic treatment effects on movement, cognition, emotion, functional disability, and quality of life. Timepoints for assessments were based on the hypothesis that delayed effects were expected.

The trial was monocentric and performed at the Center for Movement Disorders and Neuromodulation of the Heinrich-Heine-University Düsseldorf in Germany. The trial was performed according good clinicial practice, fullfiled the CONSORT criteria, was registered with ClinicalTrials.gov (NCT00902889) and approved by the local authorities according to the German Medical Devices Act (MPG), as well as by the ethics committee of the Medical Faculty of the Heinrich-Heine University Düsseldorf (3100). The study was monitored and adverse events were formally reported and evaluated by an independend data and safety monitoring board (DSMB).

### Patients

Six patients with genetically confirmed HD and predominant motor symptoms were included in the study. All patients gave written informed consent. Inclusion criteria were: symptomatic and genetically confirmed HD (CAG repeats >36) for at least 3 years, at least moderate-stage motor symptoms as measured by ≥30 points on the motor component of the Unified Huntington’s Disease Rating Scale (UHDRS) (16) and failure as measured by lack of effect or side effects with at least two medical treatments (tiapride and tetrabenazine mandatory for chorea patients) at the maximal tolerable dose. Exclusion criteria were: cognitive decline as measured by fewer than 120 points on the Mattis Dementia Rating Scale (17) or <80% of motoric performable tasks, major depression or dominant psychiatric symptoms, previous stereotactic interventions, severe brain atrophy as revealed by MRI scans (defined as cortical atrophy or atrophy of the pallidum which rendered planning of a stereotactic trajectory impossible), coagulopathy, immunosuppression, history of cerebrovascular disease, or cerebral micro- or macroangiopathy, or general medical contraindication to surgery. Inclusion and exclusion criteria were assessed twice within 3–6 months, prior to final inclusion, to ensure that included patients had a stable motoric and cognitive baseline.

### Procedure

After assessment of inclusion and exclusion criteria with a stable clinical baseline of at least 3 months (week W–1), patients underwent presurgical clinical examination. The basic examination (week W0) comprised videotaped motor functions assessed according to the UHDRS motor, chorea, and TMS-4 subscores (16), the motor scores of the Burke-Fahn-Marsden Dystonia Rating Scale (BFMDRS) (18) and the Movement Disorder Society’s Unified Parkinson’s Disease Rating Scale (MDS–UPDRS III) (19). For cognitive and mood assessment the basic test program contained the Mattis Dementia Rating Scale (MDRS) (17), the Beck Depression Inventory (BDI) (20), the Montgomery-Åsberg Depression Rating Scale (MADRS) (21), the Brief Psychiatric Rating Scale (BPRS) (22) and the Hospital Anxiety and Depression Scale (HADS) (23). An extended, detailed test program contained: the UHDRS functional/behavioral assessment, the BFMDRS disability scale, the Huntington’s Disease Activities of Daily Living (HD-ADL) (24) scale, and the Short Form Health Survey (SF-36) (25).

Surgery was performed in week 0 after baseline assesments under general anesthesia (propofol, remifentanil). Stereotactic planning was done by fusion of stereotactic CT with preoperative MRI (essentially MPRAGE, FLAIR, T2 Space). The trajectory was planned in such a way that the lowermost contact of the final electrode would be located in the upper part of the GPI, while the higher contacts would be positioned in the GPE. For intraoperative targeting, resting activity of up to five concentric oriented microelectrodes was recorded. Stimulation of the macro tip of the recording electrode above and below the target was done intraoperatively to assess possible major side effects such as stimulation of the internal capsule.

Medtronic 3387 electrodes (Medtronic Inc., Minneapolis, MN, USA) were implanted bilaterally and final electrode placement was verified by a postoperative stereotactic CT. An individualized visualization of the volume of tissue activated (VTA) was performed with a customized version of Cicerone (26), as previously described (27).

The electrodes were connected to a subcutaneous implanted Kinetra^®^ impulse generator (Medtronic Inc., Minneapolis, MN, USA). Five to seven days after surgery (week W0/1) all contacts were tested for their therapeutic range, using 120 μs pulse width and 130 Hz frequency as default settings. Stimulation was applied up to the threshold for side effects, or to the maximum of 5 V. After first testing, patients were randomized into their treatment intervention sequence: either stimulation of the two adjacent lowermost contacts (GPI) for 6 weeks, followed by stimulation of the two adjacent uppermost contacts (GPE) for 6 weeks, or vice versa. Thus three of the patients underwent the sequence GPI–GPE and three underwent the sequence GPE–GPI, during the first 12 weeks of stimulation. Stimulation was set just below the threshold for side effects but was intended to cover a broad anatomic distance of the target area and was thus chosen double monopolar. On the basis of the clinical best effect-side effect ratio, the treating physician selected either GPE or GPI stimulation for further chronic stimulation during the 6-month follow-up.

At preoperative baseline (W0) and 6-month follow-up, the detailed test battery was performed; for 6- and 12-week GPI/GPE visits, the basic test program was performed. At all timepoints tests were performed in the same order. The primary endpoints were UHDRS motor, chorea and TMS-4 scores for GPI versus GPE stimulation, and for 6 months versus baseline. Secondary endpoints were BFMDRS and UPDRS motor scores, cognition and mood scores, for GPI versus GPE and for 6 months versus baseline, as well as quality of life and functional assessments for 6 months versus baseline.

### Randomization and blinding

Patients were randomly allocated to the treatment sequence by the Coordinating Center for Clinical Trials (KKS) Düsseldorf on the basis of faxed forms filled in by the investigators. The treating physician performed the treatment, and clinical assessments of scores were performed double-blind by a scoring physician (directly for rigidity items, video rating for other motor score items) and a neuropsychologist.

### Statistical analysis

The results were analyzed by intention to treat. Data are reported as mean (SD) and compared by two-tailed paired Student’s *t*-tests. Kolmogorov–Smirnov test showed normal distribution of samples. Probability values of 0.05 or less were considered statistically significant. Comparisons were calculated for scores GPI versus GPE and baseline versus 6 months’ stimulation for primary and secondary endpoints. Additional subgroup analysis was performed excluding the Westphal variant disease subgroup. Effect size (Cohen’s *d*) was calculated for significant differences.

## Results

**Figure 1:**
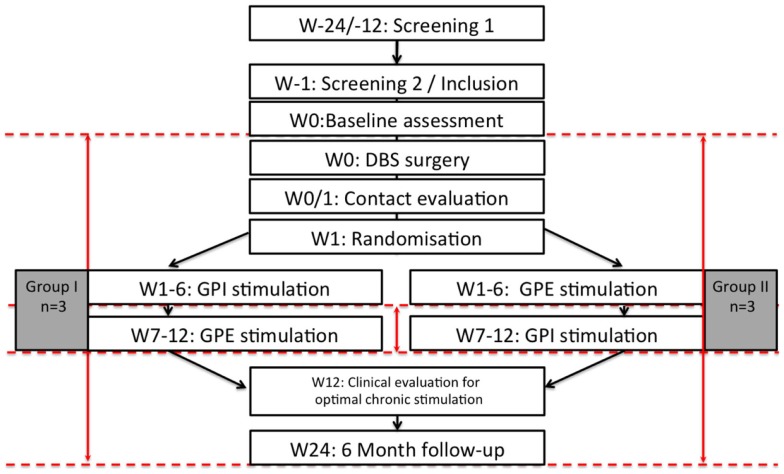
**Flow chart of the study**. *n* = 6, W: week, M: month, dashed red lines illustrate timepoints of study assessments, red arrows illustrate calculated comparisons.

**Figure 2:**
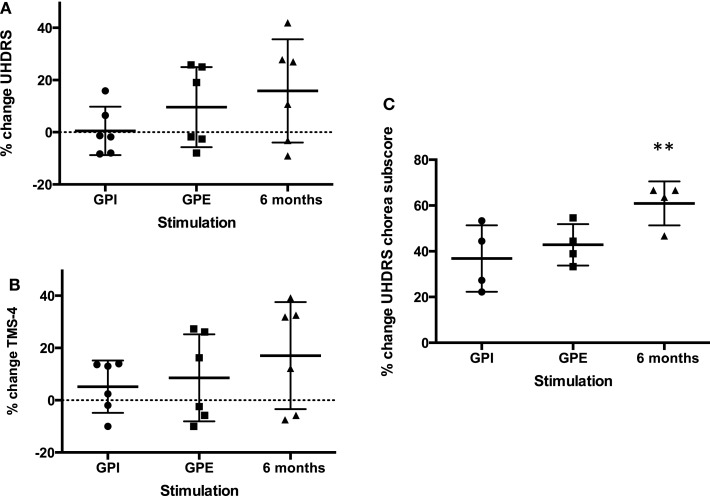
**Percent change improvement from baseline of primary endpoints**. Mean with SD (error bars) and individual results **(A)**: UHDRS, **(B)**: TMS-4, **(C)**: UHDRS chorea subscore, note: chorea subscore could only be calculated for four patients (non-Westphal). **Significant change from baseline, *p* < 0.01.

**Figure 3:**
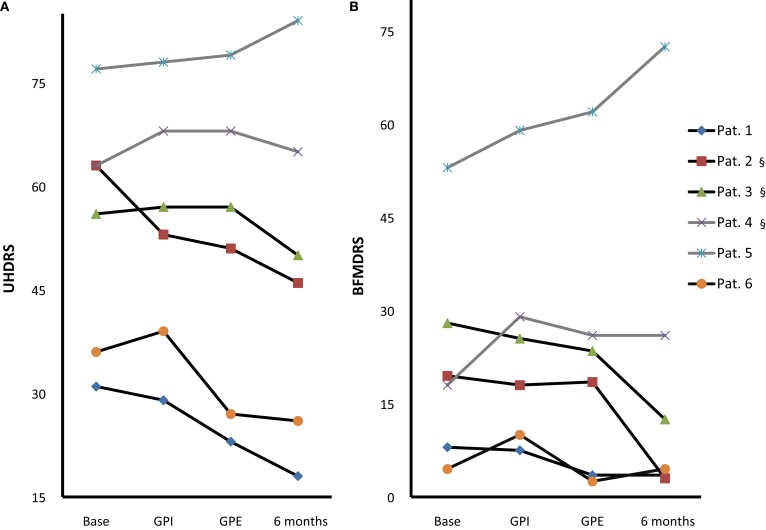
**Individual motor score changes**. Individual UHDRS **(A)** and BFMDRS **(B)** at baseline, with GPI, GPE and chronic 6-month stimulation [Pat. #1, 5, 6 GPE; Pat. #2, 3, 4 GPI (§)]; Westphal patients: gray lines, #4, 5. Note: GPI/GPE sequence was randomized with GPI first in Pat. #1, 4, 5 and GPE first in Pat. #2, 3, 6.

Six patients were included in the trial. All patients were randomly assigned, resulting in three patients receiving the intended treatment in the sequence GPE-GPI and three patients receiving the intended treatment in the sequence GPI–GPE. All patients were analyzed for the primary and secondary endpoint. For a flowchart of the study see Figure 1. Mean baseline UHDRS motor score was 54.3 with a high SD of 17.6. Two of the patients suffered from the hypokinetic-rigid Westphal variant of HD. For patient details and stimulation settings see Table 1. All patients subjectively reported improvement in daily life with GPI and GPE stimulation and at 6-month follow-up. The specific group results are reported in Tables 2–4 and illustrated in Figures 2 and 3.

**Table 1:** **A: patient characteristics and B: stimulation parameters with 120 ***μ***s and 130 Hz, two contacts “monopolar” with IPG + except Pat. #2 bipolar due to limiting side effects**.

A						

	Pat. 1	Pat. 2	Pat. 3	Pat. 4 Westphal variant	Pat. 5 Westphal variant	Pat. 6
Age (years)	52	71	38	25	23	29
Sex	M	F	M	M	M	F
Symptom duration (years)	3	21	10	11	8	4
CAG repeats	17/43	19/41	17/52	19/68	17/70	9/53
Medication (mg)	Amitriptyline (100), Trospiumchloride (60)	Sulpiride (200), Ramipril (5), HCT (25), L-Thyroxine 75 μg	Tetrabenazine (50), Tiapride (300)	Quetiapine (37.5), L-Dopa (300), Pramipexole (0.54)	Rotigotine (10), Topiramate (200), Amantadine (200), Pantoprazole (40), Domperidone (30)	Tetrabenazine (25), Tiapride (300), Citalopram (40), Quetiapine (25)

**B**							

		**Active electrodes**		**Voltage (V)**		**TEED (μJ)**	
		**Left**	**Right**	**Left**	**Right**	**Left**	**Right**

Pat. 1	GPI	0−, 1−	4−, 5−	2.5	1.8	225	108
	GPE	2−, 3−	6−, 7−	2.5	2.0	241	119
	6 Month	2−, 3−	6−, 7−	2.5	2.2	148	131
Pat. 2	GPI	0−, 1−	4−, 5+	1.5	1.7	44	50
	GPE	2−, 3−	6+, 7 −	3.0	2.7	267	79
	6 Month	0−, 1−	4−, 5+	1.5	1.7	44	51
Pat. 3	GPI	0−, 1−	4−, 5−	2.0	2.0	119	119
	GPE	2−, 3−	6−, 7−	3.6	3.6	355	521
	6 Month	0−, 1−	4−, 5−	2.0	1.5	119	90
Pat. 4	GPI	0−, 1−	4−, 5−	1.4	1.0	42	30
	GPE	2−, 3−	6−, 7−	1.7	1.7	102	51
	6 Month	0−, 1−	4−, 5−	2.4	2.4	173	173
Pat. 5	GPI	0−, 1−	4−, 5−	1.5	1.5	44	44
	GPE	2−, 3−	6−, 7−	2.0	2.0	101	101
	6 Month	2−, 3−	6−, 7−	2.0	2.0	120	92
Pat. 6	GPI	0−, 1−	4−, 5−	1.4	1.2	42	36
	GPE	2−, 3−	6−, 7−	1.5	1.3	44	39
	6 Month	2−, 3−	6−, 7−	1.5	1.3	44	39

*TEED, Total electrical energy delivered in Microjoule (μJ) per second calculated using the formula: Amplitude in Volt (V^2^) × frequency in Hertz (Hz) × pulse width in microseconds (μs) / impedance in Ohm (Ω). Mean stimulation amplitude: GPI: 1.6 V, GPE: 2.3 V, 6 months: 1.9 V*.

**Table 2:** **Motor results**.

	Baseline		GPI		GPE		6 months		*p*-Value GPI vs. GPE	*p*-Value 6 months vs. baseline
	Mean	SD	Mean	SD	Mean	SD	Mean	SD		
**A**										
UHDRS	54.3	17.6	54.0	18.1	50.8	22.2	48.2	24.4	0.181	0.160
UHDRS chorea	8.8	7.5	5.7	3.8	5.2	4.7	3.5	3.2	0.611	0.037^#^
TMS-4	37.0	12.0	35.5	13.5	35.0	15.6	32.3	15.8	0.611	0.135
UPDRS	41.3	23.3	45.5	26.1	48.7	27.9	45.8	28.3	0.066	0.117
BFMDRS	21.8	17.4	24.8	18.7	22.7	21.7	20.3	27.0	0.205	0.802
**B**										
UHDRS	46.5	15.4	44.5	12.9	39.5	17.0	35.0	15.4	0.155	0.016^##^
UHDRS chorea	13.2	4.0	8.0	1.4	7.7	3.2	5.2	2.2	0.500	0.009^###^
TMS-4	32.5	11.6	29.0	11.0	27.7	13.2	24.0	10.7	0.368	0.012^####^
UPDRS	27.2	8.8	30.5	13.8	32.5	14.0	29.0	13.6	0.343	0.523
BFMDRS	15.0	10.8	15.2	8.2	12.0	10.6	5.9	4.5	0.150	0.111

*A: complete group (*n* = 6), B: chorea subgroup, Westphal patients excluded (*n* = 4)*.

*Effect size (Cohen’s *d*): ^#^0.919, ^##^0.747, ^###^2.478, ^####^0.762*.

**Table 3:** **Results for cognition and mood: Mattis Dementia Rating Scale and subscores (in percent of performable points), BDI, MADRS, BPRS, HADS-D**.

	Baseline		GPI		GPE		6 months		*p*-Value GPI vs. GPE	*p*-Value 6 months vs. baseline
	Mean	SD	Mean	SD	Mean	SD	Mean	SD		
**A**										
Mattis Total score	88.2	7.1	86.7	8.0	86.5	9.0	86.9	7.4	0.849	0.213
Mattis Attention	88.7	6.7	89.6	6.3	89.6	7.1	86.9	7.3	1.000	0.328
Mattis Concentration	76.0	14.4	70.0	17.0	72.1	18.2	71.4	15.8	0.493	0.027^#^
Mattis Visuoconstruction	100.0^a^	0.0^a^	100.0^b^	0.0^b^	100.0^b^	0.0^b^	100.0^b^	0.0^b^	*	*
Mattis combinatoric	97.4	2.8	97.4	2.8	96.1	4.5	97.4	2.8	0.203	*
Mattis memory	89.3	7.4	87.3	13.5	85.3	11.2	90.7	7.9	0.456	0.530
BDI	6.60^c^	7.63^c^	3.00	5.02	3.83	6.15	2.60	2.70	0.402	0.230
MADRS	7.50	7.97	3.83	5.08	3.17	2.40	2.83	3.49	0.684	0.081
BPRS	26.17	8.56	21.67	4.50	21.00	2.10	21.00	3.52	0.586	0.063
HADS-D	7.60	8.08	3.50	5.82	3.50	4.59	3.60	5.68	1.000	0.04^##^
**B**										
Mattis total score	91.4	6.6	90.4	6.2	91.3	5.2	90.8	5.4	0.342	0.693
Mattis attention	91.9	5.8	91.9	6.6	93.2	4.7	91.2	4.0	0.664	0.789
Mattis concentration	82.2	13.8	77.0	15.7	82.4	10.2	79.0	13.5	0.161	0.168
Mattis visuoconstruction	100.0	0.0	100.0	0.0	100.0	0.0	100.0	0.0	*	*
Mattis combinatoric	98.7	2.6	98.7	2.6	98.1	3.8	98.7	2.6	0.391	*
Mattis memory	91.0	7.6	93.0	8.9	89.0	11.5	93.0	3.8	0.182	0.495
										
BDI	7.50	8.50	3.75	6.18	5.50	7.19	3.00	2.94	0.102	0.299
MADRS	9.00	9.76	5.00	6.00	3.25	2.99	4.25	3.50	0.432	0.249
BPRS	29.00	9.42	22.75	5.25	21.50	2.38	22.25	3.77	0.516	0.104
HADS-D	8.75	8.85	5.25	6.65	5.00	5.10	4.50	6.14	0.824	0.093

*A: complete group (*n* = 6), B: chorea subgroup, Westphal patients excluded (*n* = 4)*.

^a,b,c^Data from 3, 4, or 5 patients; *cannot be calculated.

*Effect size (Cohen’s *d*): ^#^0.304, ^##^0.573*.

**Table 4:** **Results for activity of daily living and quality of life**.

	Baseline		6 months		*p*-Value 6 months vs. baseline
	
	
	Mean	SD	Mean	SD	
**A**					
UHDRS functional capacity	12.0	8.7	12.7	9.7	0.465
UHDRS functional assessment	5.2	3.9	6.7	5.5	0.237
UHDRS behavioral assessment	8.0	4.6	4.2	5.3	0.057
HD-ADL	28.6^a^	11.0^a^	21.2^a^	16.6^a^	0.227
BFMDRS disability scale	14.2	7.8	13.0	8.9	0.302
SF-36 physical functioning	30.83	29.9	38.3	40.6	0.620
SF-36 physical role function	54.2	40.1	58.3	51.6	0.793
SF-36 bodily pain	66.7	51.6	81.7	40.2	0.648
SF-36 general health perception	63.0	21.5	79.2	12.7	0.169
SF-36 vitality	48.3	12.1	70.8	23.1	0.030^#^
SF-36 social role functioning	52.2	25.5	77.3	30.8	0.090
SF-36 emotional role functioning	72.2	44.4	77.8	40.4	0.788
SF-36 mental health	69.3	13.3	90.7	13.3	0.022^##^
**B**					
UHDRS functional capacity	16.0	7.4	17.5	7.8	0.215
UHDRS functional assessment	6.5	4.0	9.2	4.9	0.102
UHDRS behavioral assessment	8.5	4.8	5.5	6.1	0.245
HD-ADL	25.3	6.7	10.7	7.0	0.080
BFMDRS disability scale	10.2	4.7	7.7	3.9	0.030^#^
SF-36 physical functioning	42.5	30.1	57.5	35.7	0.525
SF-36 physical role function	56.2	51.5	75.0	54.0	0.319
SF-36 bodily pain	50.0	57.7	75.0	50.0	0.638
SF-36 general health perception	68.5	7.0	76.5	15.4	0.380
SF-36 vitality	47.5	13.2	66.2	28.1	0.141
SF-36 social role functioning	56.2	31.4	66.0	32.7	0.430
SF-36 emotional role functioning	58.2	50.0	75.0	50.0	0.602
SF-36 mental health	61.0	3.8	87.0	15.4	0.063

*^a^Data from five patients*.

*A: complete group (*n* = 6), B: chorea subgroup, Westphal patients excluded (*n* = 4)*.

*SF-36, UHDRS functional capacity, functional assessment: increase = improvement*.

*BFMDRS disability scale, HD-ADL, UHDRS behavioral assessment, care giver rating: decrease = improvement*.

*Effect size (Cohen’s *d*): ^#^1.22, ^##^1.609*.

### Primary endpoints: UHDRS motor score, UHDRS chorea subscore, TMS-4

#### GPI Versus GPE

Although GPE stimulation scored slightly better, there was no significant difference between GPI and GPE stimulation in terms of motor effects on the UHDRS.

#### Six Months Versus Baseline

Based on clinical judgment, three patients (#2, 3, 4) were selected for chronic GPI stimulation and three (#1, 5, 6) for chronic GPE stimulation. Analysis of the primary endpoint at 6-month stimulation versus baseline showed a mean difference of 6.1 points on the UHDRS. Due to a high SD this was not significant (for individual scores see Figure 3A). However, the UHDRS chorea subscore decreased significantly over the course of 6 months from 8.8 to 3.5 points (−5.3 (60.2%), *p* = 0.037). The effect on TMS-4 was not significant.

### Secondary endpoints

#### GPI Versus GPE

There was no significant difference between GPI and GPE stimulation in UPDRS and BFMDRS scores. Cognition and mood did not differ significantly either.

#### Six Months Versus Baseline

Effects on dystonia were not significant over the group, however half the patients (#1, 2, 3) showed marked improvement of more than 50% on the BFMDRS, with patient #2 showing a decrease from 19.5 to 3 points (−16.5 (84.7%), see Figure 3B). Further assessment of mood, cognition, functionality and quality of life revealed the following change from baseline at 6-month follow-up: cognition was stable as measured by Mattis, although a slight but significant deterioration was noted in tests of concentration, from 76.0 to 71.4% (*p* = 0.027). In accordance with the exclusion criteria, patients showed normal mood and psychiatric scores at baseline, however HADS-D (depression) improved significantly (*p* = 0.044), and MADRS and BPRS showed a statistical trend toward improvement. Several items in the quality of life and functional assessments showed significant improvement, such as the SF-36 vitality and mental health scores, while others showed a statistical trend toward improvement (SF-36 social role functioning score, UHDRS behavioral assessment).

### Subgroup analysis

*Post hoc* analysis of the choreatic subgroup of patients (non-Westphal, *n* = 4) for the primary endpoints showed no significant difference between GPI and GPE stimulation. At 6-month follow-up the primary endpoints showed significant results: the UHDRS showed a significant decrease: from 46.5 to 35.0 points (−11.5 (24.7%), *p* = 0.016). For the chorea subscore the difference between baseline and 6 months was highly significant: 13.5 compared to 5.2 points (−8 (60.6%), *p* = 0.009). The TMS-4 also showed a significant decrease, from 32.5 to 24.0 points (−8.5 (26.2%), *p* = 0.012). Concerning secondary endpoints in the non-Westphal subgroup the BFMDRS disability score improved significantly and the HD-ADL and SF-36 mental health score showed a trend toward improvement.

In the secondary motor endpoints the Westphal patients (#4, 5) showed no improvement in dystonia (BFMDRS) or hypokinetic-rigid symptoms (UPDRS) with GP stimulation.

### Medication

Medication for motor treatment was kept stable throughout the trial in most patients. In one patient (#2), sulpiride was reduced from 200mg to 100mg after the operation, while in another (#3), tetrabenazine (50mg) and tiapride (300mg) were completely withdrawn after the operation.

### Electrode localization and volume of tissue activated

**Figure 4:**
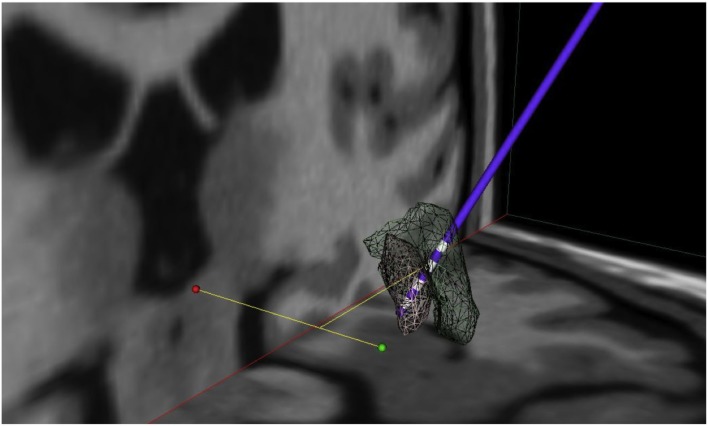
**Mean electrode localization**. Visualization of mean coordinates of left and right hemisphere mirrored to the left; 3D space relative to AC-PC line (green dot: AC, red dot: PC), gray mash: GPI, green mash: GPE; lowermost contacts comprise GPI and uppermost contacts comprise GPE stimulation. Thus, mean chronic stimulation at 6-month follow-up projects mid-electrode to the border zone between GPE and GPI. For visualization the following atlas software was used: Medtronic DBS Neurosurgical Simulator, licensed 2008, Version 1.2.3, Medtronic Inc., Minneapolis, MN, USA.

**Figure 5:**
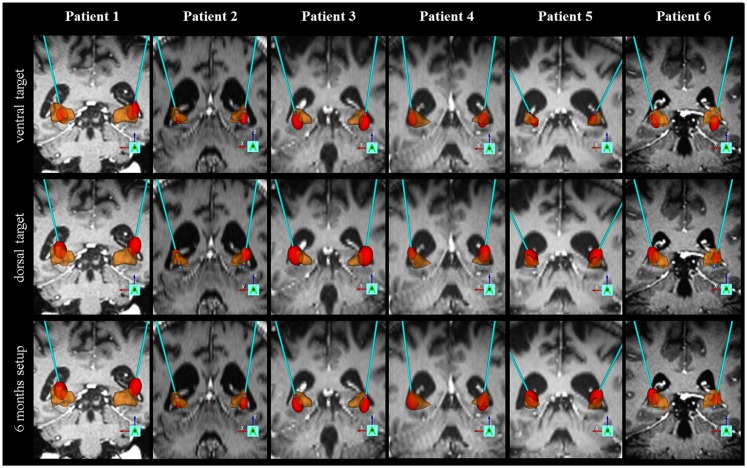
**Individual electrode localization**. Visualization on 3D coronary MRI-view of individual electrodes and volume of tissue activated (VTA, in red) in relation to the pallidum (in brown) for the ventral target (GPI), the dorsal target (GPE) and the target at 6-month follow-up.

Mean electrode localization is provided in Figure 4. Furthermore, images of individual electrodes with volume of tissue activated (VTA) are shown in Figure 5. Mean coordinates with reference to the midcommissural point [*x*, *y*, *z* (SD)] were: 21.8 (2.1), 3.8 (1.0), −3.6 (2.4) for GPI; 22.9 (2.0), 5.9 (1.2), 2.4 (3.2) for GPE; and 22.4 (2.0), 4.8 (1.8), 0.2 (5.6) for stimulation at 6-month follow-up. In summary, the mean stimulated area at 6 months was located in projection to the laminal border zone between the internal and external pallidum.

### Adverse events

No procedure-related complication such as bleeding occurred. Eight adverse events were recorded: possibly related to treatment: bradykinesia (GPI), hyperthermia possibly related to stimulation due to stable medication (GPI, Westphal), gait impairment and fall (GPI 6 month), increased chorea after reprograming due to bradykinesia (GPI 6 month); possibly related to stimulation system: deactivation of impulse generator (GPE); unrelated to stimulation but possibly due to hospitalization: thrombophlebitis (W0 postop), MRSA nose infection (W0 postop), superficial nose abrasion (GPE). In addition, two serious adverse events were reported: gait impairment and hyperkinesia after reprograming (GPI 6 month, Serious Adverse Event (SAE) criterion: leading to hospital admission and requiring reprograming) and postoperative (W0 postop) malignant hyperthermia possibly related to stimulation due to stable medication (SAE criterion: life-threatening and leading to prolonged hospital stay). All stimulation-related adverse events occurred under GPI stimulation. All adverse events resolved without sequalae.

### Individual patient discription

For individual motorscores please see Figure 3. For individual electrode localization please see Figure 5.

Patient 1 is a patient with predominant choreatic and dystonic trunk-movements that impacted his quality of life. Although the chorea sum-score was below 10 points he had benefit from DBS. Dystonic movements also improved. GPE stimulation was slightly more effective than GPI stimulation. *Patient 2* had generalized chorea. She suffered from postural instability due to anti-chorea drug treatment before surgery. With DBS chorea suppression was possible with minor impairment of balance. GPI and GPE stimulation was similar in terms of chorea reduction. GPI stimulation was better tolerated and chosen for chronic stimulation. The narrow therapeutic window between brady- and hyperkinesia remained a difficult issue resulting in two adverse events of reprograming (bradykinesia, increased chorea). Dystonia also markedly improved in this patient at 6 month follow-up. *Patient 3* showed improved chorea and dystonia, however this effect was seen mainly at 6 month follow-up. GPI stimulation was chosen for chronic stimulation. Stimulation-induced gait impairment led to a SAE in this patient. *Patients 4 and 5* suffered from the Westphal variant with strong bradykinesia and dystonia. Although objectively no improvement could be observed in the scores, the caregiver reported improved dystonia of the neck. Both Westphal patients had issues with (S)AE (pneumonia and hyperthermia). *Patient 6* had marked improvement of her generalized chorea especially with GPE stimulation. Before surgery, she had frequent falls with bone fractures due to drug treatment that could be stopped after surgery.

## Discussion

This is the first prospective trial for DBS in Huntington’s disease according to the CONSORT criteria following a predefined protocol using a controlled phase and blinded assessments of primary endpoints and with full reporting of adverse events under monitoring of a DSMB. The data provide preliminary evidence that DBS electrode implantation can be performed in a safe procedure with no procedure-related side effects. Moreover, primary endpoint analysis showed that: (1) external pallidal stimulation was equivalent to internal pallidal stimulation; and (2) chronic stimulation of the pallidum was effective in terms of significant reduction of chorea over 6 months in patients comprising large effect sizes. Secondary endpoint analysis showed that effects on dystonic symptoms varied inter-individually from no response to a strong response. Hypokinetic-rigid symptoms and Westphal patients did not improve. Cognition was generally stable over 6 months. Several measures of quality of life and functionality improved significantly, as did measures of mood. Electrode localization revealed that mean chronic stimulation at 6-month follow-up was applied in the GPI/GPE border zone.

The strengths of the current study are: the prospective design, the double-blinded assessment of the GPI- versus GPE-phase, and the examination of the effects and side-effects of chronic stimulation on non-motor aspects of HD, as well as on quality of life. On the basis of these preliminary results, it can be concluded that pallidal DBS is a potential treatment option for chorea in HD, and should be further examined in larger, multicentric, placebo-controlled trials.

Limitations of the current study should be noted. Due to the pilot, monocentric nature of the trial, sample size was limited, meaning that the finding of equivalent efficacy for GPI and GPE, in particular, could be underpowered. Furthermore, one has to be aware of the limits of statistical validity with a small sample size. Despite the small sample size for the open label comparison between baseline and GP stimulation at 6 months, we still observed remarkable reduction of chorea (60.2%), statistically however with a *p*-value (*p* = 0.037) just below the threshold of significance.

Although both the patients and the scoring physician were blinded according to the destinct stimulation site, the prospective design did not include a placebo control group, which means blinding concerning active treatment in general. At the current state, we can not rule out a bias by placebo responses and emotional state especially on chorea and quality of life. Both these limitations – the small sample size and the lack of a placebo control group – should be examined further in a larger trial. Given the authors’ experience with trials on other hyperkinetic disorders such as dystonia (2), a placebo sham stimulation controlled trial will be the next reasonable step rather than arguing in favor of an ON–OFF (crossover) design of a trial. Thus, a multi-center trial that randomizes patients directly after surgery blinded either into the stimulation ON or OFF group and assesses clinical effects after 3 months has just started. This design can control for lesion and placebo effects directly after the surgical intervention, which would not be the case for a crossover ON vs. OFF phase during the trial.

Some technical aspects of the design could have further biased the results. For safety reasons, we chose to implant only one electrode per hemisphere. Thus, more ventral “internal” and more dorsal “external” pallidal stimulation was achieved by contact programing. Although electrode targeting was adjusted accordingly, for anatomical reasons it is not possible to stimulate both the most ventral GPI and the most dorsal part of the GPE with one electrode. As we did not find it justified to implant four electrodes and in order to make the electrode position suitable for a cross-over design of GPI versus GPE stimulation, we implanted the electrodes slightly more dorsal than the usual GPI target point. One has to be aware that this approach might have biased the results. Furthermore, the fact that we used double-monopolar stimulation might have biased the spatial discrimination of effects between GPI and GPE target areas.

We calculated the mean contact position of the chronic stimulation at 6 months virtually, and visualized it on an atlas in the border zone between the GPI and GPE. Brain atrophy makes it hard to judge the exact electrode position with respect to the pallidum in HD patients. However, we furthermore provide individual electrode scans together with calculation of volume of tissue activated (VTA) of the patients.

To standardize stimulation parameters as much as possible, we worked with fixed frequencies and pulse widths. Thus, we are not able to answer questions concerning frequency effects with this study. Besides worsening of hypokinesia with high frequency stimulation of more than 130 Hz, some reports have noted improvement of chorea with minor worsening of hypokinesia at 40 Hz, suggesting that frequency settings might play a major role (10). This fits with our long-term clinical experience (28). However, there have been other reports of more heterogeneous outcomes with 40Hz (13), and even worsening of chorea (29). Besides stimulation frequency, the period of chronic application of stimulation can be of importance. We chose a period of 6 weeks of chronic stimulation to compare GPI with GPE. Although some effects on chorea could be observed within minutes, we cannot rule out that some treatment effects were missed due to the short period of stimulation. Note that several patients showed greater improvement on the UHDRS at 6 months than at any of the 6-week assessments. This observation speaks in favor of a chronic (neuroplasticity) effect of stimulation that outweighs clinical disease progression, and against a strong placebo effect. Increased treatment effect over time seems not be caused by stimulation strength as amplitudes at 6 months were similar to the mean of GPI and GPE stimulation at 12 weeks.

Concerning the generalizability of our findings, we think it is appropriate to conclude that chronic pallidal DBS should be considered as a treatment option for choreatic forms of HD. Patients without significant chorea seem not to benefit from pallidal DBS. It is, however, questionable whether our negative findings in Westphal patients can be generalized. Not only was the Westphal group too small to generate results of any significance, but the two Westphal patients suffered from the highest number of CAG repeats and the highest UHDRS scores. It is possible that the lack of effect we observed in the Westphal subgroup was due to the stage of the disease rather than to the motor phenotype (dystonia and mainly bradykinssia) itself. On the other hand, the bradykinetic phenotype might have profited more from subthalamic or posterior-ventral pallidal stimulation rather than from the chosen dorsal GPI/ventral GPE stimulation. Correlations of treatment effect with CAG repeats, UHDRS scores at baseline and burden of disease scores should be calculated in future larger trials. In the current study, the sample size was too small to allow calculation of proper correlations. A larger sample size will probably also shed light on predictors of non-response with respect to dystonic symptoms. Although the effects were not significant over the group, the current study shows that effects can be large, thus corroborating earlier findings for DBS in HD (10).

The final interpretation of our results must include a discussion of the harm-benefit ratio of this invasive treatment. It is important to note that the implantation procedure itself was safe. Adverse events were mainly related to stimulation. In line with previous findings and expected under high frequency stimulation [e.g., see Ref. (9)], we found that internal, as opposed to external, pallidal DBS can incur side effects such as gait problems and bradykinesia. Although the beneficial treatment effects did not differ significantly between GPI and GPE stimulation, the slightly larger improvement in motor scores, combined with the lower risk of side effects and higher tolerated stimulation amplitudes and TEED, seen with GPE stimulation, speak in its favor. Overall, cognition was stable across the group, however the cognitive subscore “concentration” deteriorated slightly, and no cognitive improvement was seen. In addition, due to the effect-side effect ratio, it might be reasonable to choose GPE stimulation for a larger trial, in order to try to achieve the improvement in cognition that has been reported in animal experiments (14). The results of the current trial suggest that it could also be reasonable to expect effects on quality of life in a larger trial, because even in this small sample, subscores showed significant improvement. However, as some of significant non-motor effects got lost in the non-Westphal group despite motor improvement a considerable higher number of patients will be needed to show stable results on quality of life.

In summary, it might be promising to further examine pallidal DBS concerning the question if it is an effective and safe treatment option for HD patients with severe chorea. Its effects on other outcome measures such as dystonia and non-motor aspects of the disease should also be examined in a larger trial.

## Author Contributions

LW, SG, AS, CO, SD, AR, CS, JV: study design and/or clinical management; LW, SG, SF, SE, SR, CH, MS: data acquisition of videos and clinical scores; SG: blinded video rating; LW, SF: clinical data analysis; LW, CH, JV: data analysis of electrode contacts; LW, AS, JV: drafting of the manuscript. All authors: revision and approval of the manuscript.

Members of the EHDN surgical approaches working group in preparation and during the trial were: A. Rosser – Cardiff, UK (Chair); S. B. Dunnett – Cardiff, UK (Co-Chair); J. Vesper – Düsseldorf, Germany (Co-Chair); L. Wojtecki – Düsseldorf, Germany; H. Lange – Dinslaken, Germany; C. Saft – Bochum, Germany; R. Reilmann – Münster, Germany; S. Piacentini – Florence, Italy; A. Fasano – Rome, Italy; V. Visser-Vandewalle – Maastricht, Netherlands; Y. Temel – Maastricht, Netherlands; P. Krystowiak – Amiens, France.

## Conflict of Interest Statement

Lars Wojtecki, Jan Vesper, Alfons Schnitzler, and Martin Südmeyer received consultant honoraria and travel grants from Medtronic. The remaining authors have no conflict of interest to declare.

## Abbreviations

BDI, Beck depression inventory; BFMDRS, Burke-Fahn-Marsden Dystonia Rating Scale; DBS, deep brain stimulation; GPE, globus pallidus externus, external pallidum; GPI, globus pallidus internus, internal pallidum; HADS, Hospital Anxiety and Depression Scale; HD-ADL, Huntington’s Disease Activities of Daily Living scale; HD, Huntington’s disease; MADRS, Montgomery-Åsberg Depression Rating Scale; BPRS, Brief Psychiatric Rating Scale; MDRS, Mattis Dementia Rating Scale; MDS-UPDRS, Movement Disorder Society’s Unified Parkinson’s Disease Rating Scale; SF-36, Short Form Health Survey; TEED, total electrical energy delivered; UHDRS, Unified Huntington’s Disease Rating Scale; VTA, volume of tissue activated.
